# Development of Newborn Screening for Pompe Disease

**DOI:** 10.3390/ijns6010005

**Published:** 2020-01-24

**Authors:** Wuh-Liang Hwu, Yin-Hsiu Chien

**Affiliations:** 1 Department of Pediatrics, National Taiwan University Hospital, Taipei 10041, Taiwan; chienyh@ntu.edu.tw; 2 Department of Medical Genetics, National Taiwan University Hospital, Taipei 10041, Taiwan

Pompe disease is an inborn error of lysosomal degradation of glycogen. The responsible enzyme is acid alpha-glucosidase (GAA). In the severe form of the disease, or infantile-onset Pompe disease (IOPD), weakness in the skeletal and cardiac muscles soon leads to both respiratory and cardiac failure, and death usually occurs before the age of one year. In 2006, the US Food and Drug Administration (FDA) approved Myozyme, the first drug for Pompe disease [1]. My group at the National Taiwan University Hospital (NTUH) joined the phase III clinical trial. A few patients in Taiwan were closely followed before and after they were enrolled in the trial, or an expanded-use program [2]. One patient underwent two muscle biopsies before starting treatment, and had another two biopsies thereafter. We observed progressive degeneration of the patient’s muscles. Debris-like materials filled almost all of the spaces in the myocytes in the last biopsy. Clinically, the patient gradually lost all motor abilities. We were shocked by the irreversible nature of the skeletal muscle pathologies and had hoped that the patient could have been treated earlier.

However, it is difficult for caregivers to detect muscle weakness in young infants. For example, in the Chinese culture, babies are tightly swaddled in blankets, so there are few opportunities to observe the motor developments of young infants until they can be held on their caregivers’ shoulders at an age of around 3–4 months. More often, patients with IOPD are diagnosed incidentally during respiratory infection at ages 4–5 months. Unfortunately, when signs of weakness appear, muscle damage due to glycogen storage is already extensive and irreversible. Therefore, we studied newborn screening for Pompe disease [3].

In 2006, the screening laboratory at NTUH already had experience with the in-house development of tandem mass newborn screening, and the biochemical laboratory had conducted enzymatic diagnoses of Pompe disease for more than 10 years. Two techniques, critical for Pompe disease newborn screening, were also developed at that time. The first, invented by Dr. Nestor Chamoles [4], measures lysosomal enzyme activities in punches from dried blood spots (DBS). The second was the discovery of an inhibitor, acarbose, of maltase-glucoamylase, another acid glucosidase abundant in the leukocytes [5]. Using these two methods, we measured GAA activity in DBS, eluted via overnight incubation with a fluorescence substrate. Funding is another critical element of Pompe screening. Luckily, we were able to persuade Dr. Joan Keutzer from Genzyme about the necessity of newborn screening for Pompe disease, and our ability to perform the requisite tests. Thanks to the excellent work of my senior laboratory scientist, Shu-Chuan (Sara) Chiang, and my successor, Professor Yin-Hsiu Chien, we established the protocol and proved it step-by-step. We demonstrated the screening results [6], defined the molecular epidemiology for GAA pseudodeficiency [7], proved the outcome of IOPD patients detected by screening [8], explored the features of less severe patients detected by screening [9], and then revised the screening algorithm [10]. We also added Fabry disease to Pompe disease screening using a fluorescence substrate [11]. However, true multiplex newborn screening was made possible only after the development of tandem mass substrates by Dr. Michael Gelb at the University of Washington [5,12].

Soon after, several pilot programs, including programs in Italy, Australia, Japan, Korea, USA, and Hungary, have tested the feasibility of Pompe newborn screening and understood the incidence of Pompe disease and the impact of the disease. After the Discretionary Advisory Committee on Heritable Disorders in Newborns and Children (DACHDNC) added Pompe disease to the Recommended Uniform Screening Panel (RUSP) in 2013, the spread of Pompe disease newborn screening increased. However, challenges remain, including sensitivity and specificity of the assays, management of pseudodeficiency, time and method to treat IOPD patients detected by screening, immunomodulation, and management of later-onset Pompe disease patients discovered by screening. In this special issue of the *International Journal of Neonatal Screening*, global experiences with Pompe disease newborn screening were pooled to enhance the understanding of screening and improve the outcomes of patients affected by Pompe disease.
